# ROS-triggerable dual nanocoated probiotics for inflammatory bowel disease treatment

**DOI:** 10.1080/10717544.2026.2630460

**Published:** 2026-02-14

**Authors:** Guangze Sang, Sizhen Wang, Qiwei Tai, Yunchang Zhang, Xufang Wang, Baoling Yan, Weiwei Jiang, Zhendong Chen, Linhong Sun, Jiao Zhou, Xiaoxian Wu, Zi Ye, Feng Yang, Jun Luo, Beibei Guo

**Affiliations:** a School of Pharmacy, Naval Medical University, Shanghai, People's Republic of China; b School of Pharmacy, Fujian University of Traditional Chinese Medicine, Fuzhou, Fujian, People's Republic of China

**Keywords:** Inflammatory bowel disease, ulcerative colitis, probiotics, catechin, *Escherichia coli* Nissle 1917

## Abstract

Inflammatory bowel disease (IBD), an immune-mediated chronic gastrointestinal disease, is difficult to treat because its specific pathogenic mechanisms are currently unclear, and its recurrence rate is high. Probiotic therapies have been used to treat IBD; however, challenges, including poor digestive stability and unstable treatment effects, remain. Catechins are natural flavonoids with anti-inflammatory and anti-oxidant properties. In this study, we link catechins to the amino side chain of ethylene glycol chitosan through phenylboronic acid as a nano coating for probiotics, and enhance their stability under acidic conditions with sodium alginate. The resulting double-layer nano coating system is triggered by reactive oxygen species (ROS) for probiotic-small molecule combination therapy. After oral administration, the nano coating helps probiotics stabilize and pass through the harsh digestive environment, respond to the high ROS environment at the site of inflammation, and release anti-inflammatory drugs. The coating breaks down and adheres to the cell surface while releasing probiotics to regulate the intestinal microbiota environment and achieve combined therapy. The method exerts a certain therapeutic effect in a dextran sulfate sodium (DSS)-induced mouse model of ulcerative colitis. This study combines traditional targeted drug therapy concepts with biological therapies to improve IBD treatment efficacy and reduce side effects.

## Graphical abstract

**Figure:**
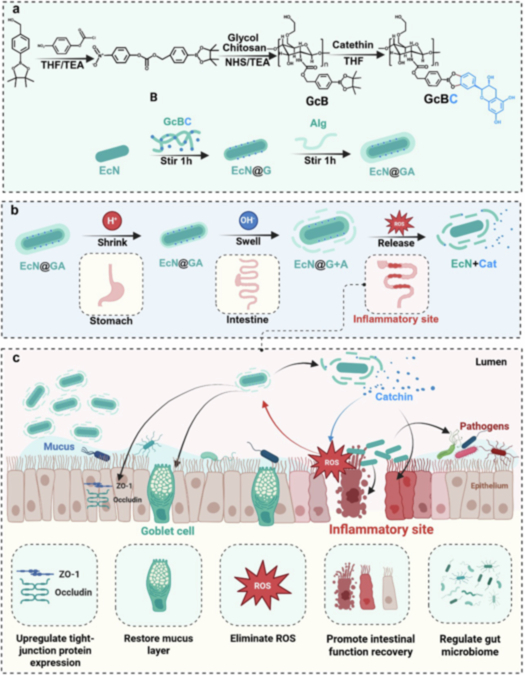

## 1. Introduction

Inflammatory bowel disease (IBD) is an immune-mediated chronic disease with no cure, and it significantly impacts patient quality of life. IBD can be subdivided into Crohn's disease (CD) and ulcerative colitis (UC) (Torres et al. 2017; Ungaro et al. 2017). Gender differences, genetic factors, lifestyle, and living environment all affect the occurrence and development of IBD (Ananthakrishnan et al. 2022; Kammermeier et al. 2023; Andersen et al. 2024). Moreover, due to increases in disease incidence, treatment costs are also increasing (Burisch et al. 2023). The main clinical symptoms include abdominal pain, diarrhoea, bloody stools, and low-grade fever. Current treatment drugs include non-steroidal anti-inflammatory drugs, immunosuppressants, monoclonal antibodies, Janus kinase inhibitors, and antibiotics (Al-Bawardy et al. 2021; Virtanen et al. 2024). These drugs have many side effects, are difficult to tolerate after long-term use, and cannot completely cure IBD (Núñez F et al. 2022; Núñez et al. 2023).

In recent years, research has shown that the gut microbiota plays an important role in alleviating and advancing IBD (Zhang et al. 2022). Faecal microbiota transplantation and probiotic supplementation are now available treatment options (Khoruts and Sadowsky 2016; Ji et al. 2023; Bethlehem et al. 2024). *Escherichia coli* Nissle 1917 (EcN), a well-known probiotic strain discovered almost a century ago, plays an important regulatory role in the gut microbiota and has mature applications in the treatment of IBD, antibiotic-associated diarrhoea, and other related conditions (Zhao et al. 2022; Chen et al. 2023; Bai et al. 2023). However, because of differences in the gut microbiota and immune systems between individuals, the therapeutic effect of probiotics is not as stable or reliable as that of traditional therapeutic drugs in clinical practice. The harsh digestive environment poses a serious challenge to the efficacy of probiotic therapies (Ji et al. 2023). Recently, various probiotic modification technologies have been developed to improve the efficacy of probiotic therapies, and this area of research is showing increasing promise (Wu and Liu 2022).

The identification of target drugs in natural products is also being explored in relation to IBD. One major characteristic of IBD is the presence of significant inflammatory changes at intestinal lesion sites, which significantly elevate the levels of inflammatory mediators such as reactive oxygen species (ROS) and tumour necrosis factor-*α* (TNF-*α*) (van Loo and Bertrand 2022), and increase tissue permeability (Torres et al. 2017; Ungaro et al. 2017). Polyphenol compounds in plants have been widely researched in relation to anti-inflammatory and antioxidant therapy; their catechol structures are used in various structural modifications, and polyphenol compounds also have an important regulatory effect on intestinal microorganisms (Jamieson et al. 2023; Han et al. 2023; Vaghari-Tabari et al. 2024).

**Figure 1.**
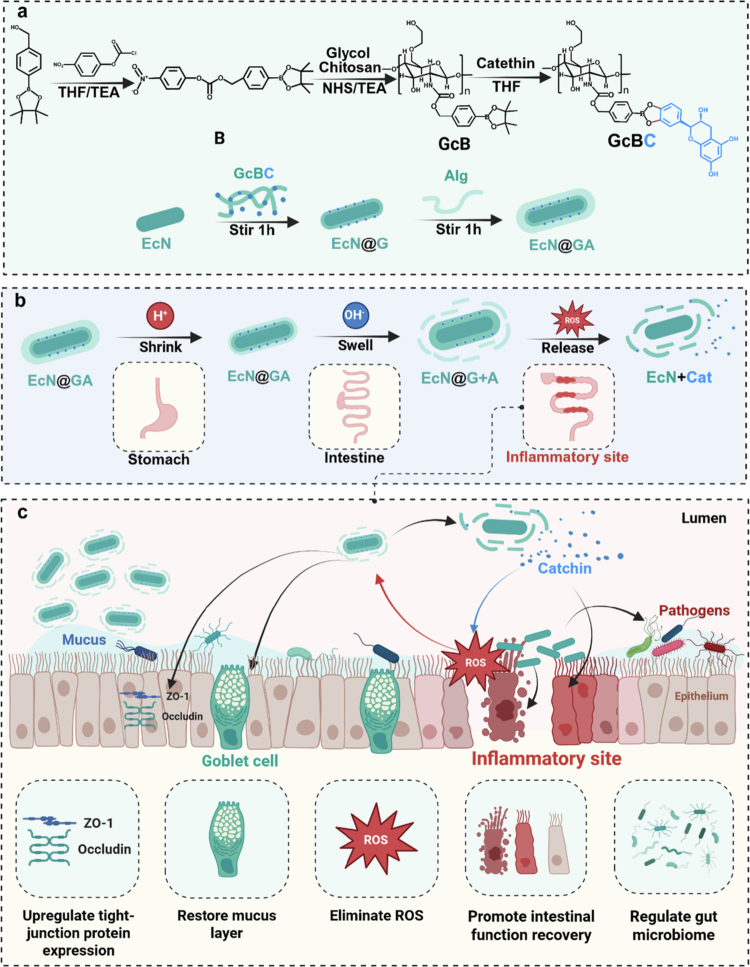
Schematic showing the construction and treatment mechanism of an ROS-triggerable dual nanocoated probiotic. a: Preparation of ROS-triggerable dual nanocoated probiotic. b: ROS-triggerable combination therapy. c: Combination therapy mechanism for the treatment of IBD. Created using https://BioRender.com.

Based on the above considerations, we chose catechin (Cat) as a therapeutic molecule for research and transformation (Fan et al. 2017) and to produce a combined therapeutic effect with probiotics. As a highly efficient anti-inflammatory and antioxidant small molecule (Bernatoniene and Kopustinskiene 2018). Cat may play a positive role in treating IBD (Fan et al. 2017; Bernatoniene and Kopustinskiene 2018). The high ROS environment at the site of inflammation can also serve as a triggering factor for targeted delivery systems (Hartwig et al. 2021). In this study, a ROS-responsive protective shell loaded with Cat (Figure 1a) was designed to deliver probiotics for the treatment of IBD.

## 2. Materials and methods

### 2.1. Reagents and cells

Glycol chitosan (Gc, degrees of polymerisation = 400, Cat no: G810700), 5-aminosalicylic acid (5-ASA, Cat no: A823148), 5-fluoresceinisothiocyanate (FITC, Cat no: I6141), rhodamine B isothiocyanate (RBITC, Cat no: R817238), and 1,1'-dioctadecyl-3,3,3',3'-tetramethylindotricarbocyanine iodide (DIR, Cat no: D864067) were obtained from Shanghai Macklin Biochemical Technology Co., Ltd. (Shanghai, China). Catechin (Cat, Cat no: BD1457), dextran sulphate sodium (DSS, molecular weight = 40000, Cat no: BD123894), 4-hydroxymethylphenylboronic acid pineal ester (Cat no: BD218088), and *p*-nitrophenyl chloroformate ester (Cat no: BD34896) were purchased from Bideparm, Inc. (Shanghai, China). Tetrahydrofuran (THF, Cat no: 14018AD), Triethylamine (TEA, Cat no: 17471 H), *N*-hydroxysuccinimide (NHS, Cat no: 69489 C), dimethyl sulfoxide (DMSO, Cat no: 75927 K), methanol (Cat no: 75851 V), ethanol (Cat no: 73537AQ), petroleum ether (Cat no: G5500322B), and sodium alginate (Cat no: 89186F) were purchased from Adamas Inc. (Shanghai, China). All cell culture reagents were purchased from Adamas Life, Inc. (Shanghai, China). CCK-8 cell kits (Cat no: C0005) were purchased from TOPSCIENCE Co., Ltd. (Shanghai, China). Dialysis membranes (molecular weight cut-off (MWCO): 3500 Da, Cat no: MD34-3.5) were purchased from Viskase® Companies, Inc. (Lombard, Illinois). 5,5-dimethyl-1-pyrroline *N*-oxide (DMPO, Cat no: D048) was purchased from DOJINDO Chemical Technology Co., Ltd (Shanghai, China).

*Escherichia coli* Nissle 1917 (NCBI SN: Z_CP022686.1) was purchased from Beijing Biobw Biotechnology Co. Ltd. (Beijing, China). Caco-2, L929, and HT-29 cells were purchased from Hefei All Thing Biological Technology Co. Ltd. (Hefei, China). C57BL/6 female mice, SPF grade (18–22 g) were purchased from Hangzhou Ziyuan Experimental Animal Technology Co., Ltd. (Zhejiang China) and housed at 20–24 °C and humidity <60%.

### 2.2. Synthesis of ROS responsive linker phenylboronic acid ester (B)

4-Hydroxymethylphenylboronic acid pinal ester (2.5 g) was dissolved in 100 mL of THF, TEA (2225 μL) was then added, and stirred until dissolved. *p*-Nitrophenyl chloroformate (2.35 g) was added and stirred for 24 h. Ethyl acetate was added to terminate the reaction, extract with saturated NaHCO_3_ and take the organic components, spinned to evaporate and obtain the crude product. The developing agent was prepared based on the ratio of petroleum ether: ethyl acetate (4:1), eluted on a silica gel column, and spun to evaporate and obtain the target product (Shen et al. 2021).

### 2.3. Synthesis of GcB

Gc (40 mg) was weighed, dissolved in TEA (0.3 mL), added to water (15 mL), and stirred. Next, B (40 mg) and NHS (11.5 mg) were dissolved in THF (15 mL), dropped into the above Gc-TEA-water solution, stirred for 12 h, washed with methanol three times, dialysed, and freeze-dried in deionized water containing 2% methanol to obtain GcB (Shen et al. 2021).

### 2.4. Synthesis of GcBC

GcB (45 mg) was weighed, deionized water (19 mL) and THF (3.75 mL) were added, and the mixture was stirred until dissolution. Cat (15 mg) was dissolved in THF (1 mL), and the solution was dropped into the previously prepared solution, stirred at 25 °C for 24 h, dialysed with a deionized water and methanol solution (deionized water: methanol = 50:1) for 48 h, freeze-dried, and GcBC was obtained (Shen et al. 2021).

### 2.5. Preparation of the fluorescent-labelled shell

To prepare FITC-GcBC, GcBC was dissolved in 0.5 M sodium carbonate buffer (2 mg/mL), and FITC was dissolved in a 0.5 M sodium carbonate solution (1 mg/mL). The FITC solution was then added dropwise to the Gc solution at a volume ratio of 5:1. The solution was stirred at 25 °C in the dark overnight and dialysed using water and a cellulose membrane (MWCO 3500) for 2 d. FITC-GcBC was obtained by freeze-drying in the dark (Son 2003).

### 2.6. Preparation of RBITC-Alg

An Alg solution (2 mg/mL) with a pH of 8.0 was prepared. The RBITC/DMSO solution (1 mg/mL) was added to the Alg solution at a volume ratio of 5:1. The mixture was stirred overnight at 25 °C in the dark, and ethanolamine was added to terminate the reaction. A cellulose membrane (MWCO 3500) was used for 2 d for water dialysis, followed by freeze-drying to obtain RBITC-Alg (Mladenovska et al. 2007).

### 2.7. Characterisation of the ROS-response drug loaded shell

The ^1^H NMR spectrum of each material was acquired using a Bruker instrument (AVANCE NEO 600 MHz). The FTIR spectra of each material were characterised using an FTIR instrument (Bruker TENSOR II). UV-Vis spectra were recorded on an Agilent Cary 60. The zeta potential and particle size measurements were performed using a Malvern Zetasizer Nano apparatus. Mass spectra were acquired using an Agilent UPLC-QTOF/MS (Agilent 1290 Infinity-6538 UHD and Accurate-Mass QTOF/MS). EPR data were recorded using a Bruker EMX PLUS.

The antioxidant activity of GcBC was also evaluated using chemical methods, and the total antioxidant activity was determined using an ABTS assay kit, according to the manufacturer's instructions (Munteanu and Apetrei 2021). The classic ortho phenanthrene method was used to evaluate the **·**OH elimination ability of GcBC. Orthophenanthrene forms a stable orange-red complex with divalent Fe ions. The Fenton reaction generates **·**OH to oxidise divalent iron, and the trivalent iron formed by the oxidation of divalent iron forms a colourless complex with ortho phenanthrene. The absorption peak at 536 nm disappeared, and the antioxidant capacity of the sample was evaluated by detecting changes in absorbance (Munteanu and Apetrei 2021).

### 2.8. Drug loading rate and encapsulation efficiency of GcBC

GcBC was dissolved at a concentration of 1 mg/mL, and UV absorbance at 280 nm was measured after centrifugation at 10000 rpm for 30 min. The UV absorbance at 280 nm of the supernatant was then measured again. DLC and EE were calculated according to the following equations:

(1) $$\mathrm{DLC}\left(\%\right)=\frac{\mathrm{Total weight of Cat in GcBC}}{\mathrm{Total weight of GcBC}}\times 100\%$$

(2) $$\mathrm{EE}(\%)=\frac{\mathrm{Total}\;\mathrm{weight}\;\mathrm{of}\;\mathrm{encapsulated}\;\mathrm{Cat}\;\mathrm{in}\;\mathrm{GcBC}}{\mathrm{Total}\;\mathrm{weight}\;\mathrm{of}\;\mathrm{Cat in}\;\mathrm{GcBC}}\times 100\%$$

### 2.9. Drug release evaluation *In vitro*

GcB (5 mg) and GcBC (5 mg) were added to DMSO-d6 (600 μL) with or without H_2_O_2_ (30% wt, 6 uL) solution. The mixture was dissolved and mixed thoroughly, and the ^1^H NMR spectrum was recorded after a specific reaction time.

GcBC (1 mg/mL) was dissolved in PBS, placed in a dialysis bag (MWCO 3500), and immersed in PBS solution (20 mL) containing a specific concentration of H_2_O_2_. The resulting solution (100 μL) was taken for quantitative detection by UV-Vis spectrophotometer at a specific time point at 280 nm. An equal amount of PBS solution was added to the dialysis bag, and each sample was tested in triplicate.

### 2.10. Cell cytotoxicity assay

L929 and HT-29 cells were cultured in high-glucose Dulbecco’s modified Eagle’s medium (DMEM) containing 10% foetal bovine serum (FBS), whereas caco-2 cells were cultured in MEM (containing 10% FBS and NEAA). The cells were then seeded into a 96-well plate (5000 cells/well), incubated at 37 °C overnight. The medium was removed, and high glucose DMEM (100 μL) and MEM (100 μL) medium containing the sample but without FBS were added to each well. The concentrations of Gc, GcB, GcBC, Cat, and 5-ASA were adjusted to 25, 50, 100, 200, 400, and 800 μg/mL, respectively. After 24 hours of incubation, the culture medium was removed, and 100 μL of 10% CCK-8 serum-free medium was added, incubated for 4 hours, and then the UV absorbance was measured at 450 nm.

### 2.11. Construction of EcN@GA

This method was based on a previously reported method with the required adjustments (Anselmo et al. 2016; Zhou et al. 2022). GcBC was dissolved in deionized water to prepare a 1 mg/mL solution. EcN was centrifuged to remove the LB medium (LB), dissolved in deionized water, and diluted to an optical density value of 0.5 at 600 nm (OD_600_). GcBC solution with EcN solution were stirred to mix (100 rpm) then centrifuged (3000 rpm, 5 min) to remove the supernatant and washed twice with deionized water. Alg was dissolved in deionized water to prepare a 1 mg/mL solution and coated using the same method to obtain EcN@GA.

### 2.12. Characterisation of EcN@GA

After fixing with glutaraldehyde, the bacteria were subjected to gradient dehydration with ethanol and observed under a scanning electron microscope (SEM, ZEISS GeminiSEM 300). An appropriate concentration of the fixed bacteria/ethanol solution was dropped onto a carbon-coated Cu mesh. The sample was naturally dried in clean air, and its morphology was observed using transmission electron microscopy (TEM, FEI Talos F200X, UK). DLS was used to measure the zeta potential and particle size changes in EcN before and after encapsulation. After coating the surface of EcN with the synthesised fluorescently labelled shell, confocal laser scanning microscopy (CLSM) was used to observe the coating conditions, and a flow cytometer (BD CytoFLEX) was used to quantitatively detect the changes in fluorescence intensity, with the detection wavelengths set at 525 nm (FITC) and 585 nm (RBITC).

### 2.13. Evaluation of *In vitro* growth of probiotics

EcN and EcN@GA were diluted with LB medium to an OD_600_ value of 0.2 and incubated at 37 °C. The OD_600_ value was then recorded every 30 min for 8 h.

### 2.14. *In vitro* tolerance capability

To evaluate the stability of probiotics in the digestive environment, EcN, EcN@G, and EcN@GA were incubated in PBS, LB, SGF, SIF, and 4% BS solutions at 37 °C and 200 rpm, respectively. The OD_600_ values were recorded at specific time points.

### 2.15. Cell adhesion ability

HT-29 cells were diluted (8000 cells/mL), cultured for 24 h to adhere to a dish, incubated at 37 °C for 30 min with 2 μM DIR staining solution, and then washed with PBS. EcN and EcN@GA coated with fluorescently labelled shells were diluted to an OD_600_ value of 0.5. HT-29 cells were incubated for 2 h, washed twice with PBS, and observed under a confocal microscope (Zeiss LSM 800).

### 2.16. *In vitro* ROS scavenging ability

The total antioxidant capacity was tested using a commercial ABTS kit (Adamas, cat. C8315-300T-PKG) according to the manufacturer’s instructions. A gradient concentration sample solution was prepared according to the manufacturer’s instructions. After 3 min of reaction, the absorbance was measured at 405 nm, and the total antioxidant capacity was calculated.

The pyrogallol auto-oxidation method was used to test the **·**O_2_–scavenging ability of the probiotics. The pyrogallol solution, including 0.90 mL Tris-HCl buffer and 0.05 mL pyrogallol solution (60 mM) was prepared to generate **·**O_2_^–^, then 0.05 mL sample solution (1 mg/mL) was added to the pyrogallol solution. The absorbance was recorded at 325 nm using a UV-Vis spectrophotometer every 30 s for 5 min.

Electron paramagnetic resonance (EPR) was used to detect ROS clearance. In summary, photocatalytic hydrogen peroxide can generate **·**OH and **·**O_2_^–^ (Nosaka and Nosaka 2017; Yang et al. 2022; Yang et al. 2022). After adding an antioxidant, some ROS can be cleared, and DMPO can be used to capture uncleared ROS. The ROS scavenging ability was evaluated based on the peak height of the EPR spectra.

### 2.17. DSS-induced colitis mouse model

A total of 37 mice were used in this study, including 6 for intestinal adhesion research section, 25 for therapeutic effects research section, and 6 for biosafety research section, using ear studs to label mice. After one week of adaptive feeding, the mice were provided 3% DSS water solution for free consumption and normal food for 7 days. The successful construction of a mouse UC model was based on the observation of reduced activity, mental fatigue, diarrhoea, rectal bleeding, and weight loss, as well as positive results in occult blood tests (Wirtz et al. 2017).

### 2.18. Therapeutic assessment of the UC mouse

The mice were randomly divided into five groups of five mice: healthy control, DSS, 5-ASA, EcN, and EcN@GA. After modelling, each group received the corresponding treatment for 7 days. The healthy and DSS groups were orally administered with 0.2 mL of PBS per day, whereas the 5-ASA group was orally administered with 0.2 mL of 5-ASA water solution per day (100 mg/kg). EcN and EcN@GA were orally administered EcN and EcN@GA strains at a concentration of 1 × 10^8^ CFU. On the second day after the end of treatment, mice were sacrificed to evaluate the therapeutic effects.

### 2.19. Weight and disease activity index (DAI)

Daily changes in body weight, diarrhoea, and rectal bleeding were recorded for each group of mice. The data were used to calculate the DAI according to a previous study (Praveschotinunt et al. 2019).

### 2.20. *In vivo* intestinal adhesion evaluation

An IVIS imaging system (PerkinElmer IVIS Lumina III) was used to evaluate the rotation time of the probiotics. 6 mice were randomly divided into two groups, after the oral administration of 1 × 10^8^ CFU of Cy7-labelled EcN and EcN@GA, fluorescence images were captured at specific times (Feng et al. 2020).

### 2.21. Organ index and colonic oedema status

The spleen, liver, and colon were harvested immediately after sacrifice. The organ indices were calculated as the organ weight-to-body weight ratio. Colonic oedema status was calculated as colon weight (mg)/colon length (cm). A high colonic oedema index indicates severe colitis.

### 2.22. Enzyme linked immunosorbent assay (ELISA)

All mouse blood samples were collected using the orbital blood collection method, with each mouse being sampled once and a volume of 0.8-1ml collected per mouse. After natural settling for 4 h, the serum was separated by centrifugation at 2000 rpm for 10 min. An ELISA kit was used to detect TNF-*α* in the serum (Kuang et al. 2023).

### 2.23. Immunofluorescence staining

Colon tissue was fixed in 4% paraformaldehyde for 24 h and embedded in paraffin for sectioning. After dewaxing, staining was performed, and cells were incubated overnight with ZO-1 and Occludin primary antibodies, washed three times with PBS (pH 7.4), incubated with secondary antibody for 1 h, incubated with DAPI for 10 min, washed three times with PBS (pH 7.4), and observed under a fluorescence microscope. Tunel staining was performed according to the manufacturer’s instructions.

### 2.24. Histopathology

Colon samples were fixed with 4% paraformaldehyde for 24 h, embedded in paraffin, and subjected to H&E and AB/PAS staining to observe tissue damage and mucosal layer recovery.

### 2.25. Microbiome analysis

The intestinal contents of the mice were collected after different treatments and frozen in liquid nitrogen. DNA was extracted, and PCR amplification was performed. Based on the amplification results, a library was established and compared with a bacterial gene library to calculate the microbiota abundance of each sample. Alpha and beta diversity were analysed, and differences in the microbiota were compared (Chen et al. 2025).

### 2.26. *In vivo* biosafety research

C57BL/6 female mice (6−8 weeks) were randomly divided into two groups. After the oral administration of PBS or EcN@GA for 7 days, the mice were sacrificed on the 10th day, and blood samples were collected for routine blood and biochemical testing. The main organs were collected for hematoxylin and eosin staining.

### 2.27. Statistical analysis

All data are expressed as mean ± standard error of mean (SEM), and statistical analysis is performed using one-way analysis of variance (ANOVA) and Tukey’s test, statistical significance is expressed as *P < 0.05, **P < 0.01, ***P < 0.001, and ****P < 0.0001. Bioinformatics analysis was performed using OmicStudio tools https://www.omicstudio.cn/tool and the TUTU analysis platform (https://www.cloudtutu.com/).

## 3. Results and discussion

### 3.1. Preparation and characteristics of the ROS-response drug-loaded shell

Phenylboronic acid ester linkers, known for their exceptional ROS responsiveness and ease of modification, have been extensively used in ROS-responsive drug delivery systems (Qiu et al. 2017; Wang et al. 2021). In high-ROS environments, they undergo cleavage and release their loaded cargo. Phenylboronic acid esters were synthesised using a two-step method. The catechol structure of Cat forms a five-membered ring with a boronic acid section, and the synthesis pathway is described in Figure 1a.

**Figure 2.**
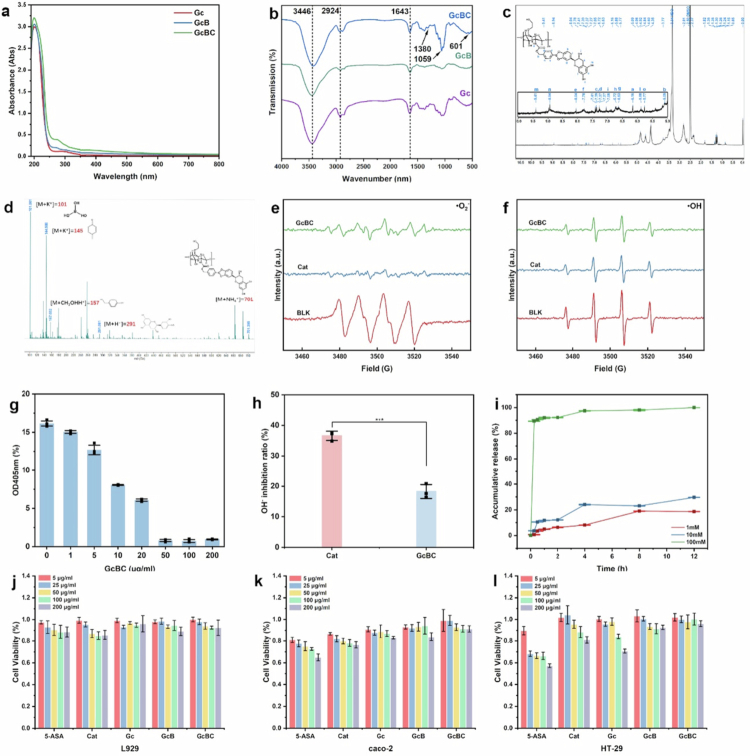
Structural, antioxidant, ROS-responsive and biocompatible characterisation of the ROS-response drug-loaded shell. a, b: UV-Vis and FT-IR spectrum of Glycol chitosan (Gc), Glycol chitosan- Phenylboronic acid ester (GcB), and Glycol chitosan- Phenylboronic acid ester -Catechin (GcBC). c, d: ^1^H NMR and mass spectrum results for GcBC. e, f: Electron paramagnetic resonance (EPR) results for the **·**OH and **·**O_2_^–^ elimination ability of GcBC. g: ABTS method to evaluate the total antioxidant ability of GcBC. h: 1, 10-phenanthroline method was used to evaluate the **·**OH elimination ability of GcBC. **i**: ROS-responsive accumulation release profile of GcBC. j, k, l: Cell viability of L929, caco-2 and HT-29 cells treated with 5-aminosalicylic acid (5-ASA), Cat, Gc, GcB, and GcBC. Data are expressed as the mean ± standard error of the mean (SEM, *n* = 3 for g−i, *n* = 6 for j−l, biological replicates).

The synthesised product was characterised using UV-Vis and FT-IR spectroscopy. The drug loading capacity (DLC, %) of Glycol chitosan- Phenylboronic acid ester -Cat (GcBC) was 17.8 ± 0.8%, and the encapsulation efficiency (EE, %) was 49.4 ± 2.0%, respectively. After modifying the amino groups of the chitosan side chains with Cats, the absorption at 280 nm increased significantly (Figure 2a), and the stretching vibration absorption peak of the C-O bond appeared at 1643 cm^−1^. The stretching vibration peak of the hydroxyl group and the absorption peak of the free amino group in the main chain overlapped at 3446 cm^−1^, forming a wide absorption band; the methylene structure in the six membered ring formed a sharp shoulder peak at 2924 cm^−1^; After introducing the phenylboronic acid ester structure, boric acid formed a stretching vibration peak at 1380 cm^−1^, and the carbon-carbon double bond structure in the benzene ring appeared at 601 cm^−1^. The characteristic absorption peak of Cat shifted to approximately 1059 nm, which was significantly enhanced in the GcBC group, confirming the successful synthesis of GcBC (Figure 2b).

We employed ^1^H NMR to evaluate the materials, assigning peaks to each and focusing primarily on peak variations within the range of 5–10 ppm. The synthesised product was characterised by ^1^H NMR spectroscopy. **Figure S1** shows the ^1^H NMR spectrum and proton peak assignment of the phenylboronic acid ester. Similarly, compared with Glycol Chitosan (Gc) **(Figure S2),** the synthesis of Glycol chitosan- Phenylboronic acid ester (GcB) (**Figure S3)** and Glycol chitosan- Phenylboronic acid ester -Catechin (GcBC) (Figure 2c) was characterised using ^1^H NMR spectroscopy.

After treatment with 1 mM H_2_O_2_ for 30 min, the characteristic peaks of GcB at 7.34, 7.78, and 8.0 (**Figure S4)** and the characteristic peaks of GcBC disappeared (**Figure S5),** indicating that the phenylboronic acid ester group could be oxidised and cleaved by H_2_O_2_. This confirmed the successful synthesis of GcB and GcBC.

Mass spectrometry was performed on the synthesised B, GcB, and GcBC, and the ion peaks of B, GcB, and GcBC and the corresponding oxidation products were separated and detected, further confirming the successful synthesis of each component (Figure 2d, S6-S7).

The study on the antioxidant capacity of GcBC using Electron paramagnetic resonance (EPR) and chemical methods showed that GcBC can reduce the peak height of·OH and ·O_2_^–^ (Figure 2e-f). The ABTS method showed that the total antioxidant capacity of GcBC increased with increasing concentration. In contrast, the ortho-phenanthrene method indicated that the antioxidant capacity of GcBC was lower than that of Cat at the same concentration (Figure 2g-h).

### 3.2. *In vitro* ROS-responsive drug release

We used a UV-Vis spectrophotometer to measure the release of Cat. The phenylboronate side chain is oxidatively broken, and Cat is released into the solution under a hydrogen peroxide environment. As anticipated, the release of GcBC in a PBS solution containing hydrogen peroxide (1, 10, and 100 mM) was 3.4%, 10.5%, and 90.5% within 0.5 h, and the release rates gradually increased with increasing hydrogen peroxide concentration and time (Figure 2i).

### 3.3. Cell viability

The cytotoxicity of GcBC was evaluated using the CCK-8 assay with L929, HT-29, and caco-2 cells. After incubation with materials of different concentrations for 24 h, the 5-aminosalicylic acid (5-ASA) and Cat groups showed significant cell inhibitory effects with increasing concentrations. However, the GcBC group did not exhibit significant cytotoxicity, which may be explained by the relatively low level of free Cat, most of which were linked to Gc by B.

Cell viability staining showed that incubating cells at 200 μg/mL resulted in the highest cell death rate in the 5-ASA group. The GcBC group had a similar proportion of live and dead cells as the normal group, indicating that GcBC had low cytotoxicity (Figure 2j−l).

### 3.4. Preparation of EcN@GA

EcN naturally carries a negative charge on its surface, whereas Gc carries a positive charge owing to its free amino group branches and sodium alginate (Alg) carries a negative charge because of its carboxyl groups. Using a layer-by-layer method (Anselmo et al. 2016), Gc and Alg can be individually encapsulated and assembled via electrostatic interactions. In complex gastrointestinal environments, both can ensure that a sufficient number of EcNs reach the disease site and release Cat in a responsive manner in high ROS environments, synergistically producing therapeutic effects (Figure 1a–b).

**Figure 3.**
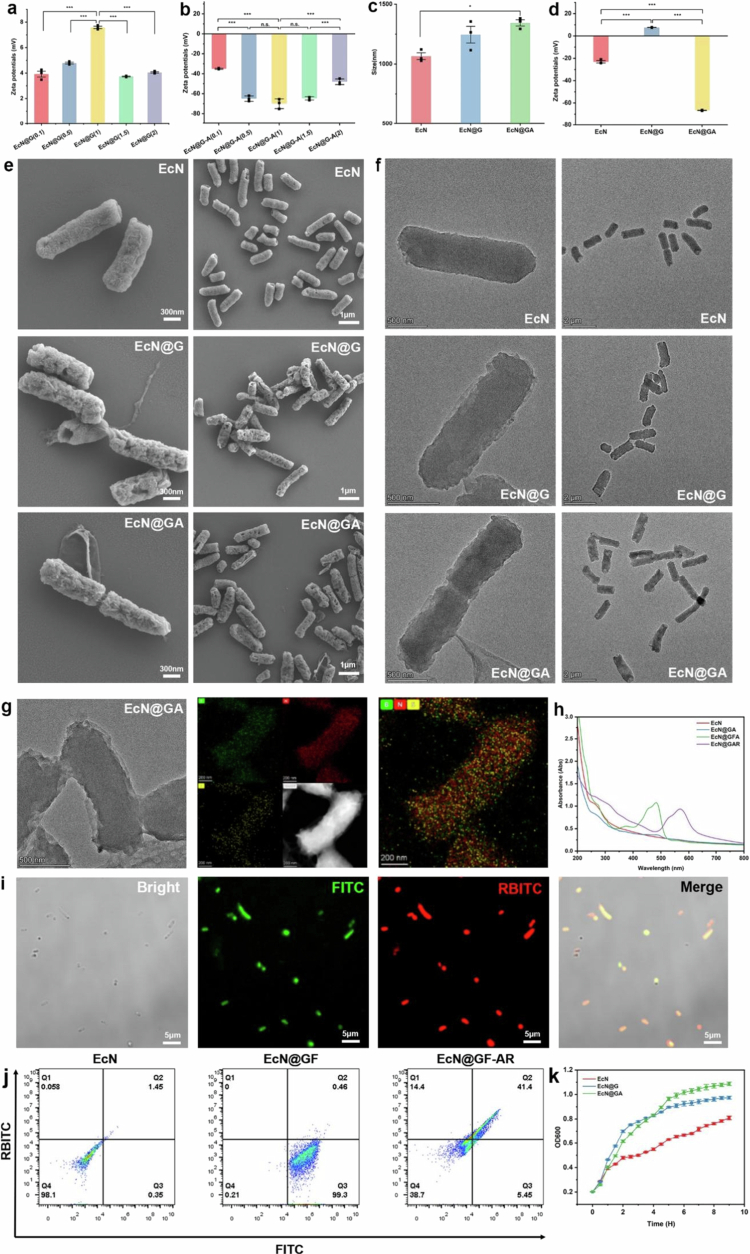
Characterisation of physicochemical properties, morphology and growth performance of GcBC/Alg nanocoated Escherichia coli Nissle 1917 (EcN). a, b: Zeta potential characterisation of EcN encapsulated with different concentrations of GcBC and sodium alginate (Alg) (from 0.1 to 2 mg/mL). c: Size distribution of nanocoated EcN. d: Zeta potential reverses during the encapsulation process. e, f: scanning electron microscope (SEM) and transmission electron microscopy (TEM) images of EcN, EcN@G, and EcN@GA. g: Element mapping of EcN@GA. h: UV-VIS spectrum results of uncoated and coated EcN. i: confocal laser scanning microscopy (CLSM) images of EcN@GFAR. The green channel represents 5-fluoresceinisothiocyanate (FITC) labelled GcBC and the red channel represents rhodamine B isothiocyanate (RBITC) labelled Alg. j: Flow cytometric analysis of EcN, EcN@GF, and EcN@GFAR. k: Growth curves for EcN, EcN@G, and EcN@GA. Data are expressed as the mean ± standard error of the mean (SEM, *n* = 3, biological replicates), analysed by one-way ANOVA, followed by Tukey’s test (n.s., not significant, *P < 0.05, **P < 0.01, ***P < 0.001).

Different concentrations of GcBC and Alg were tested to determine the optimal concentration for coating the bacteria (Figure 3a–b). As the concentration changed, the absolute value of the zeta potential was the highest when both the GcBC and Alg concentrations were 1 mg/mL. Therefore, this concentration was used to coat the bacteria in subsequent experiments.

To confirm the successful encapsulation of EcN, we measured the changes in the zeta potential and particle size after coating with different layers. Owing to their significant charge differences, the reversal of the zeta potential indicated the successful encapsulation of the shell. The average particle size of bacteria on the dynamic light scattering detection surface increased slightly after being applied as a shell coating (1065.6 nm to 1343.6 nm Figure 3c). The zeta charge can undergo significant charge reversal after being coated with different shell layers (−23.1 mV, 7.59 mV, −66.7 mV), which can be explained by the charge changes caused by the encapsulation of shells with different properties (Figure 3d).

scanning electron microscope (SEM) and transmission electron microscopy (TEM) images show that EcN@GA has a rough nanoshell, which is significantly different from naïve EcN, forming a clear contrast with the smooth and clear cell walls of naïve bacteria (Figure 3e–f). Energy dispersive spectrometry (EDS) results showed that the boron in the nano coating overlapped with the bacterial nitrogen and sulphur and was detectable at the periphery, indicating that the nano coating was successfully applied to the bacterial surface (Figure 3g) (Zheng et al. 2024).

To further demonstrate the successful encapsulation of GcBC and Alg, we synthesised 5-fluoresceinisothiocyanate (FITC)-labelled GcBC (GF) and rhodamine B isothiocyanate (RBITC)-labelled Alg (AR) (Son 2003; Mladenovska et al. 2007). After coating with different fluorescent-labelled nano layers, the UV spectrum of EcN showed absorption peaks at 483 and 569 nm (Figure 3h). Confocal laser scanning microscopy (CLSM) and flow cytometry were performed. The CLSM results showed that the FITC/RBITC fluorescence signal was co-localised with the bacteria (Figure 3i), indicating that the GcBC and Alg shells were successfully coated on the bacterial surface. After coating with different shell layers, the increase in fluorescence intensity was quantitatively confirmed by flow cytometry, which further verified the results. The number of particles with high-fluorescence intensity in the modified bacteria was significantly higher than that in the naive bacteria (Figure 3j). In summary, these results indicate that the presented method and materials can be used to successfully coat the surfaces of probiotics (X. Yang et al. 2022; Han et al. 2024).

### 3.5. Characterisation of coated probiotics

To determine whether the encapsulation process affected bacterial growth, we recorded the growth conditions of EcN, EcN@G, and EcN@GA in LB medium. All three strains exhibited similar growth curves in LB medium (Figure 3k). Under neutral or alkaline conditions, the carboxyl side chains of sodium alginate exist in an ionised form with a negative charge. Electrostatic repulsion occurs between negatively charged carboxyl groups, causing the molecular chains to stretch and form a loose network structure. Under acidic conditions, the charge on the carboxyl side chains of sodium alginate changes, resulting in a change in the intermolecular forces, a decrease in electrostatic repulsion, and a relative increase in hydrogen bonding and other interactions between the molecular chains. The molecular chains approached and aggregated to others, resulting in a compact network. Similarly, owing to the presence of amino side chains, the chitosan microstructure undergoes changes opposite those of sodium alginate. In neutral or alkaline environments, the amino group loses hydrogen ions, and the repulsion between adjacent molecular chains with the same charge increases, resulting in the coating having a loose and broken molecular structure. This principle has been used to design pH-responsive protective coatings that enable probiotics to reach the colon (Wang et al. 2022; Parsana et al. 2023).

**Figure 4.**
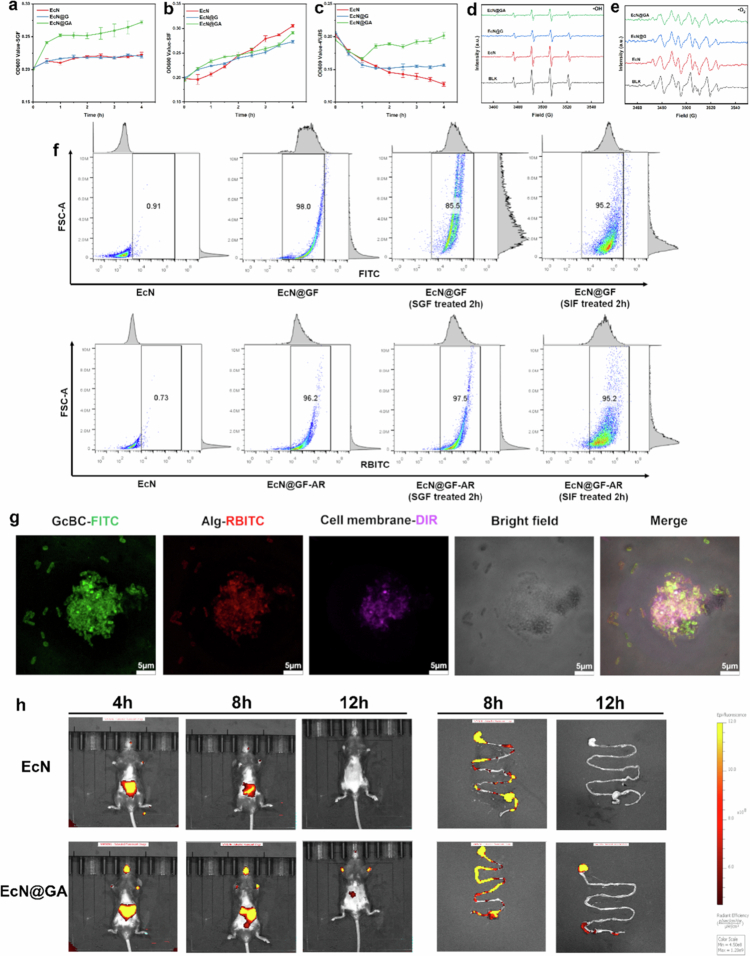
Enhanced survival and adhesion of coated *Escherichia coli* Nissle 1917 (EcN). a, b, c: Growth curves of EcN, EcN@G, and EcN@GA in simulated gastric fluid (SGF), simulated intestinal fluid (SIF), and 4% bile acid salt (4% BS). d, e: Electron paramagnetic resonance (EPR) results of the **·**OH and **·**O_2_^–^ elimination ability of EcN, EcN@G, and EcN@GA. f: Flow cytometry results of single and dual nanocoated EcN treated with SGF and SIF. g: confocal laser scanning microscopy (CLSM) image of EcN@GA adhesion to HT-29 cells. h: IVIS images of mice after oral administration of uncoated/coated EcN. Data are expressed as the mean ± standard error of the mean (SEM, *n* = 3, biological replicates).

We studied the survival rate of probiotics in patients treated with simulated gastric fluid (SGF), simulated intestinal fluid (SIF), and 4% bile acid salt (4% BS). In the SGF/4% BS environment, EcN and EcN@G showed similar growth curves, whereas the growth curve of EcN@GA was significantly better, indicating that the protective ability of a single GcBC coating on bacteria was limited under acidic conditions, and the presence of an alginate coating was necessary. In the SIF environment, the growth curves of the three groups were similar, indicating that the alginate nano coating had a limited effect on bacterial growth (Figure 4a–c).

EPR was used to determine the ability of EcN@GA to eliminate ROS. In short, the photocatalytic hydrogen peroxide method was used to prepare ·OH and ·O_2_^–^. After the addition of the different samples, the remaining ROS captured by 5, 5-dimethyl-1-pyrroline *N*-oxide (DMPO) were detected. Compared with the blank, the EcN@G and EcN@GA groups showed better ROS scavenging ability, and the difference between the EcN@G and EcN@GA groups was not significant (Figure 4d–e). These results indicate that the ROS scavenging ability of EcN@GA originated from the GcBC coating, and the Alg coating had no significant effect.

We further investigated the protective effect of the nano coating using flow cytometry. After 2 h of SGF incubation, the volume of the particles in the EcN@GF suspension was significantly increased, and the particle size of EcN@GFAR was slightly increased. After 2 h of SIF incubation, both particle sizes increased significantly, confirming our conclusion (Figure 4f).

Inspired by a previous study (Abdi et al. 2021) and considering that the intestinal inflammatory site is usually positively charged (Hartwig et al. 2021), it is hypothesised that the negatively charged alginate in the outer layer of probiotics may aid in accurate adherence to the inflammatory site and the achievement of a targeted therapeutic effect. We investigated the effects of nano coatings on the adhesion ability of bacterial cells. The dual-layer coating was labelled with FITC and RBITC, whereas the HT-29 cells were labelled with the cell membrane-staining dye 1,1'-dioctadecyl-3,3,3',3'-tetramethylindotricarbocyanine iodide (DIR). After co-incubation for 2 h, EcN@GAs adhered to the surrounding cells in large quantities (Figure 4g).

To further investigate the retention characteristics of probiotics in mice intestines, we administered EcN and EcN@GA labelled with Cy7 dye. The results showed that 4 h, 8 h and 12 h after oral administration, EcN@GA exhibited a stronger fluorescence intensity, indicating that the nano coating helped prolong the retention time of probiotics in the intestine (Figure 4h, S8) (Liu et al. 2021; Wang et al. 2024; Guan et al. 2025).

### 3.6. Therapeutic effects of EcN@GA against DSS-induced colitis in Mice

**Figure 5.**
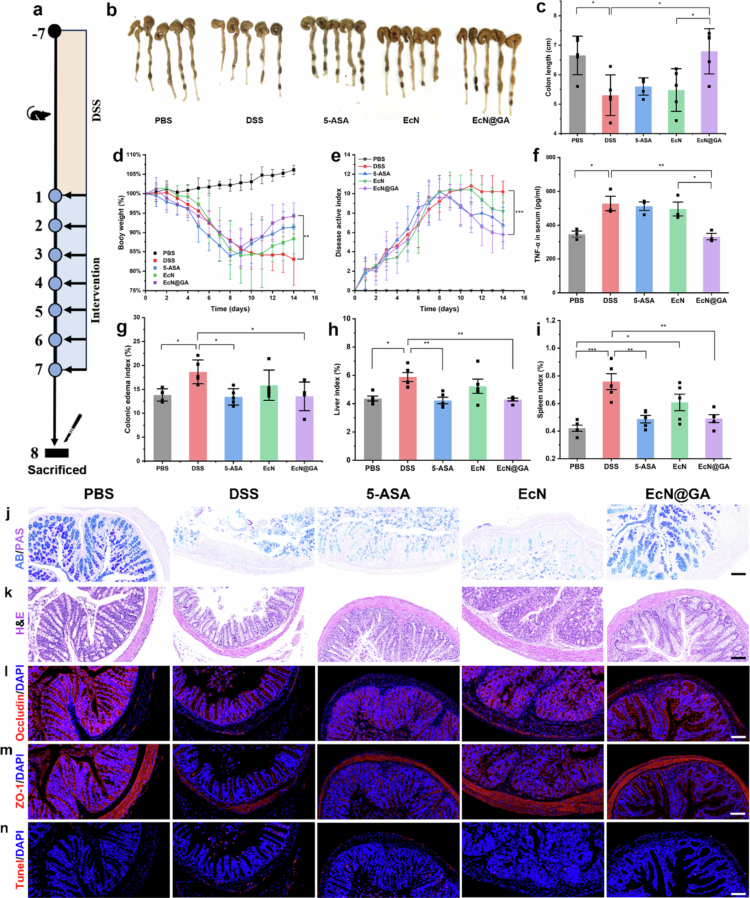
Therapeutic effects of EcN@GA against DSS-induced colitis in mice. a: Schematic of the modelling and treatment procedure used for the mouse experiments. b, c: Colon images and length of mice in different treatment groups. d, e: Weight and DAI changes during modelling and treatment. f: necrosis factor-*α* (TNF-*α*) in the serum measured by ELISA. g, h, i: Colonic oedema, liver, and spleen indices for each group at the end of the treatment. j, k: Representative AB/PAS and H&E staining images of colon tissues, scale bar: 50 μm. l, m, n: Representative immunofluorescence images of occluding, ZO-1, and Tunel in the colon. The red channel represents ZO-1, Occludin, and apoptotic intestinal cells, whereas the blue channel represents cell nuclei stained with DAPI. Scale bar: 50 μm. Data are expressed as the mean ± standard error of the mean (SEM, *n* = 5, biological replicates), analysed by one-way ANOVA, followed by Tukey’s test (n.s., not significant, *P < 0.05, **P < 0.01, ***P < 0.001).

Based on the aforementioned data, the therapeutic effects of the coated probiotics were evaluated in a mouse model of DSS-induced colitis. After 7 days of DSS modelling, drug or probiotic treatment was administered for 7 consecutive days (Figure 5a). Mice were sacrificed on day 8, and the colon was collected (Figure 5b). In accordance with our research findings, during the modelling period, the body weight of all mice exhibited a pronounced decline, whereas the DAI increased significantly (Figure 5d–e). Nevertheless, following treatment with either medication or probiotics, there was a variable degree of recovery, and the DAI diminished. However, external disease manifestations in the PBS group remained unchanged. The extent of weight recovery was similar between the EcN@GA and normal groups.

Mouse serum was used for ELISA to elucidate the alterations in the inflammatory factors, as depicted in Figure 5f. Subsequent to EcN@GA treatment, TNF-*α* was substantially diminished, signifying an amelioration of the mouse inflammatory status.

The mice were then euthanized by cervical dislocation, and the colon, liver, and spleen were collected and measured. The colonic oedema index was calculated as the ratio of colon length to mouse body weight. In contrast, colonic oedema and organ indices in the DSS group were significantly increased, whereas colonic length was significantly reduced, indicating the occurrence of inflammatory symptoms (Wu et al. 2022). In contrast, the results for the EcN@GA group showed that the degree of intestinal inflammation in mice decreased after treatment. Colon tissue samples were harvested for further investigation (Figure 5c, g–i).

The H&E and AB/PAS staining outcomes of the colon demonstrated that in the PBS group, the intestinal crypts disappeared, the intestinal epithelial and goblet cells were disrupted, and showed the most severe damage, and the fundamental internal structure of the intestine was almost entirely lost. In contrast, in the other groups, goblet cells showed a variable degree of recovery, and the intestinal tissue in the EcN@GA group was relatively well preserved. The restoration of the mucus layer in the EcN@GA group was more conspicuous, as evidenced by the greater quantity of blue-stained mucus proteins (Figure 5j–k).

ZO-1 and Occludin are intestinal tight junction proteins that play crucial roles in maintaining intestinal integrity and normal physiological processes. Tunel staining was used to detect cell apoptosis in intestinal tissue, and immunofluorescence staining was conducted for colon samples from each group. The results revealed that the expression of ZO-1 and Occludin in the EcN@GA group was restored, the intestinal structure was recovered, and the number of apoptotic cells was markedly lower than that in the PBS group, indicating that this system contributed to the maintenance of normal expression levels of intestinal tight junction proteins and the reduction of cell apoptosis. (Figure 5l–n).

### 3.7. Regulatory effects of EcN@GA on gut microbiota

**Figure 6.**
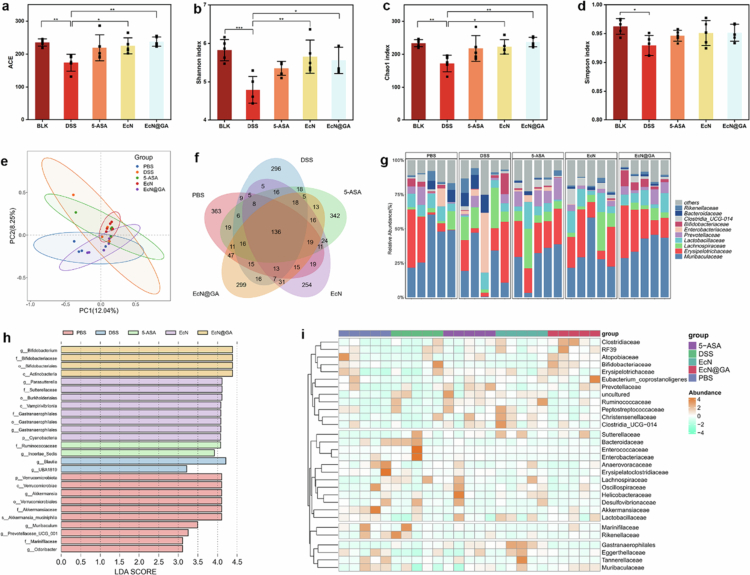
Regulatory effects of probiotics on the intestinal microbiome. Abundance-based coverage estimator (ACE, a), Shannon diversity (b), Chao1 diversity (c), and Simpson diversity (d) indices of mice after different treatments. e, f: PCoA analysis results and Venn diagrams for each group. g: Relative abundances of the intestinal microbiome at family levels in mice. h: LefSe analysis of each group. i: Heatmap of the relative abundances of the top 30 families in the gut microbiota. Data are expressed as the mean ± standard error of the mean (SEM, *n* = 5, biological replicates), analysed by one-way ANOVA, followed by Tukey’s test (n.s., not significant, *P < 0.05).

An imbalance in the gut microbiota is often closely related to IBD, and the composition of the gut microbiota in patients with IBD is significantly different from that in healthy individuals (De Vos et al. 2022; Zhang et al. 2022). One of the goals of emerging gut microbiota transplantation therapies is to restore a normal gut microbiota composition (Shan et al. 2022). We further investigated the regulatory effect of EcN@GA on the gut microbiota of DSS-induced UC mice. *α*-diversity analysis results showed that the ACE, Shannon index, and Chao1 index of DSS modelling group were higher than healthy group, EcN group and EcN@GA. The Simpson index of the healthy group is higher than DSS modelling group. Indicated an increase in gut microbiota diversity in mice after treatment (Figure 6a–d). After treatment, the proportion of Bifidobacteria was significantly higher than in the other groups.

In the *β*-diversity analysis, we used the Jaccard algorithm to analyse PCoA maps for each group (Figure 6e). The difference between the treatment and healthy groups was smaller, indicating that the gut microbiota of treated mice was closer to that of healthy mice. The differences between each group are represented by a Venn diagram (Figure 6f).

Linear discriminant analysis (LDA) effect size (LefSe) results showed that the differences of each group. Under normal circumstances, there are significant differences in the gut microbiota environment between patients with inflammatory bowel disease and normal individuals, which has been confirmed in our experimental results. Specifically, Akkermansia, a signature microbiota of healthy intestines, is more abundant in healthy mice than in other groups, and this bacterium can maintain intestinal barrier integrity by degrading mucus layer components (Gu et al. 2021; Zhao et al. 2025) After treatment with EcN@GA, the abundance of *bifidobacteria* in the group is higher than that in other groups, indicating EcN@GA can increase the probiotic level in the gut of mice. Based on literature research, this bacterium, as a core anti-inflammatory probiotic in IBD treatment, can effectively alleviate inflammatory responses associated with inflammatory bowel disease (IBD) by regulating the intracellular JNK signalling pathway, improving DSS-induced intestinal epithelial barrier dysfunction, and differentially modulating the expression levels of inflammatory factors such as TNF-*α* (Strisciuglio et al. 2015; Sato et al. 2021). On the other hand, the abundance of *Blautia* is increased in the DSS model group. Relevant studies suggest that the elevated abundance of this bacterium is associated with inflammatory progression (Motiani et al. 2020; Lee et al. 2023). These findings indicate that EcN@GA can significantly improve the intestinal microbiota composition of ulcerative colitis (UC) mice by regulating the balance between pro-inflammatory and anti-inflammatory bacteria. The heatmap also showed significant differences in the composition of the strains among the groups (Figure 6g–i). These findings indicate that EcN@GA greatly improved the composition of the intestinal microbiota in UC mice.

### 3.8. *In vivo* biosafety evaluation

After validating the therapeutic effects of EcN@GAs, we evaluated their biosafety *in vivo*. Mice were divided into two groups (*n* = 3). After intragastric administration for 7 days, the mice were sacrificed on the tenth day, and their blood and major organs were collected for further examination. The major organs (liver, kidney, and spleen) and blood indices were determined. As expected, there were no significant differences in the blood indices (**Figure S9a-m**) and histomorphology (**Figure S9n**) of the major organs between treated and untreated mice. These results demonstrate that EcN@GA has good biosafety.

## 4. Conclusions

Probiotics generally exhibit less consistent and stable efficacy than traditional chemical drugs and biological antibodies for the treatment of clinical IBD (Zhang et al. 2022; Mousa and Al Ali 2024; Qiao and Zhang, 2025). In this study, we successfully constructed a small-molecule probiotic combination treatment system. Probiotic-loaded drugs can be released in high-ROS environments at the site of inflammation. Encapsulated probiotics have good resistance to the digestive environment, as well as stronger anti-inflammatory, antioxidant, and gut microbiota regulation abilities, without affecting normal growth characteristics. Catechins were loaded onto glycol chitosan side chains using ROS-responsive linkers, and EcN was encapsulated with sodium alginate via electrostatic interactions. Catechin not only exerts a therapeutic effect but also enhances the survival of probiotics in the intestine. The retention time of catechin was also extended due to the intestinal retention properties of the probiotics. After oral administration, EcN@GA exerted a certain therapeutic effect in a DSS-induced mouse model. Strategies that combine probiotics with traditional therapeutic drugs represent a promising direction for future research with broad prospects for clinical applications. Compared with traditional bulk encapsulation technologies, single bacterial encapsulation technologies can more accurately control the thickness of the encapsulated material, provide a wider range of material selection, and the survival rate of bacterial strains is higher (Lin et al. 2023; Geng et al. 2023; Wang and Liu 2024). This method was adopted in this study, however, it has certain limitations. The experimental steps are more complicated than bulk encapsulation method, and require the consideration of numerous control conditions.

## Supplementary Material

Revised SI.docx Revised SI.docx

## Data Availability

Data supporting the findings of this study are available from the corresponding author upon request.
