# Supplementary material for: ROS-triggerable dual nanocoated probiotics for inflammatory bowel disease treatment
Source: Drug Deliv. 2026 Feb 14;33(1):2630460. doi: 10.1080/10717544.2026.2630460 (PMC12912239; doi:10.1080/10717544.2026.2630460)
Supplement: Revised SI.docx [file IDRD_A_2630460_SM1545.docx]

Supporting Information

ROS-Triggerable Dual Nanocoated Probiotics for Inflammatory Bowel Disease Treatment

Guangze Sang^1, #^, Sizhen Wang^1, #^, Qiwei Tai^1, #^, Yunchang Zhang^1, #^, Xufang Wang^1^, Baoling Yan^2^, Weiwei Jiang^1^, Zhendong Chen^1^, Linhong Sun^1^, Jiao Zhou^1^, Xiaoxian Wu^1^, Zi Ye^1^, Feng Yang^1,2^, Jun Luo^1,*^, Beibei Guo^1,*^


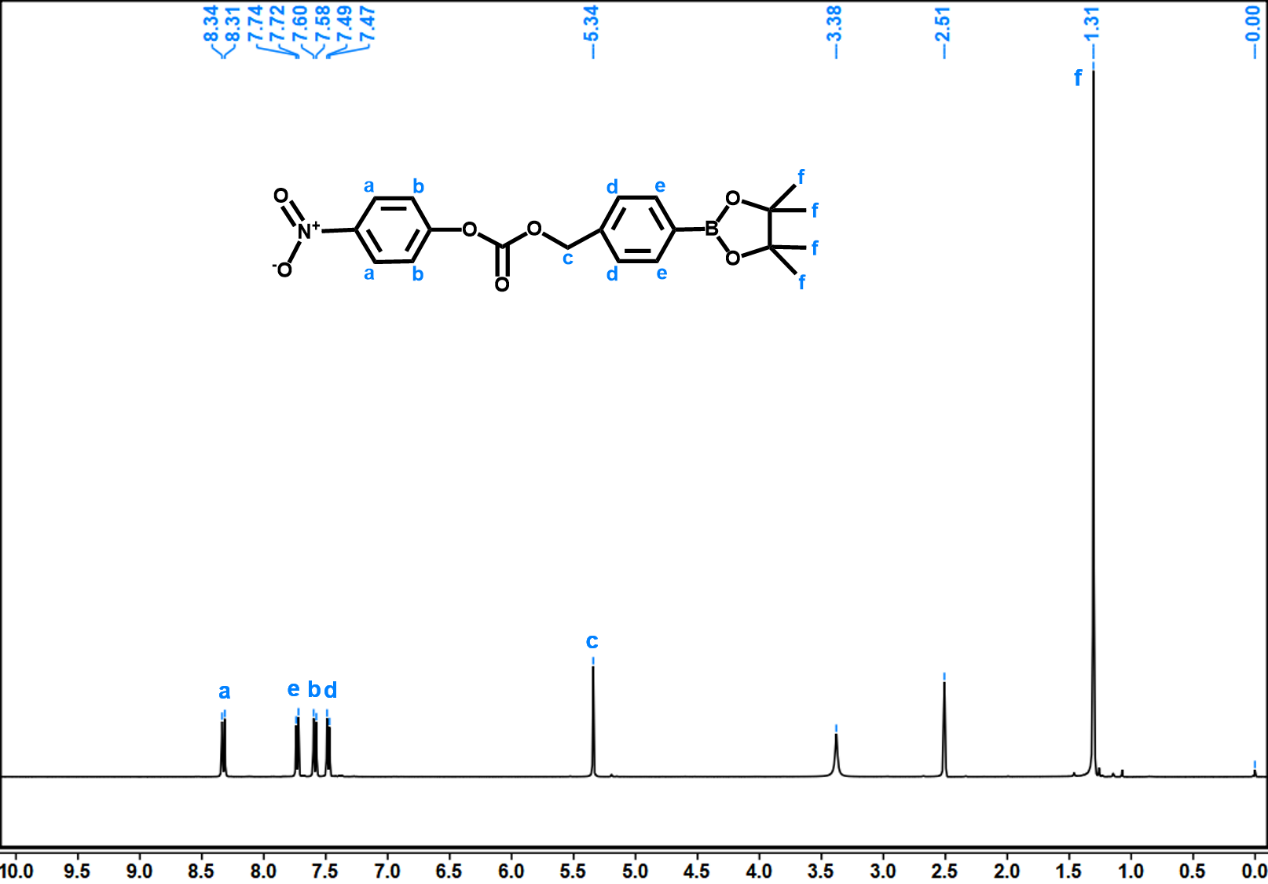


**Figure S1.** ^1^H NMR spectrum results for phenylboronic acid ester (B).


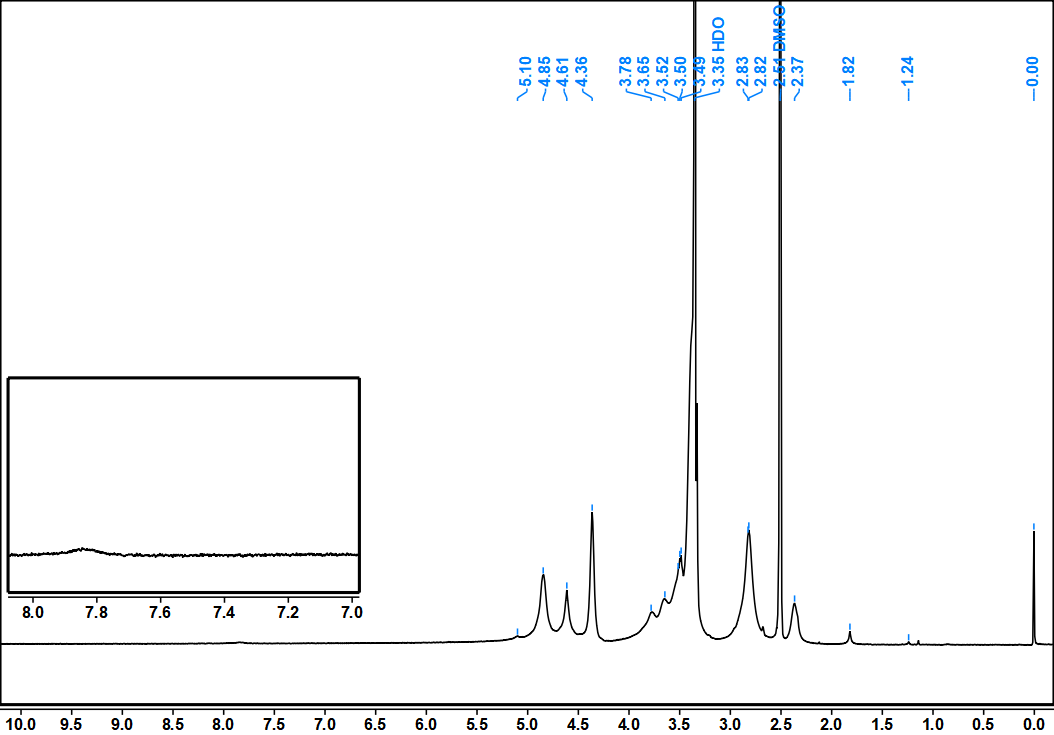


**Figure S2.** ^1^H NMR spectrum results for Gc.


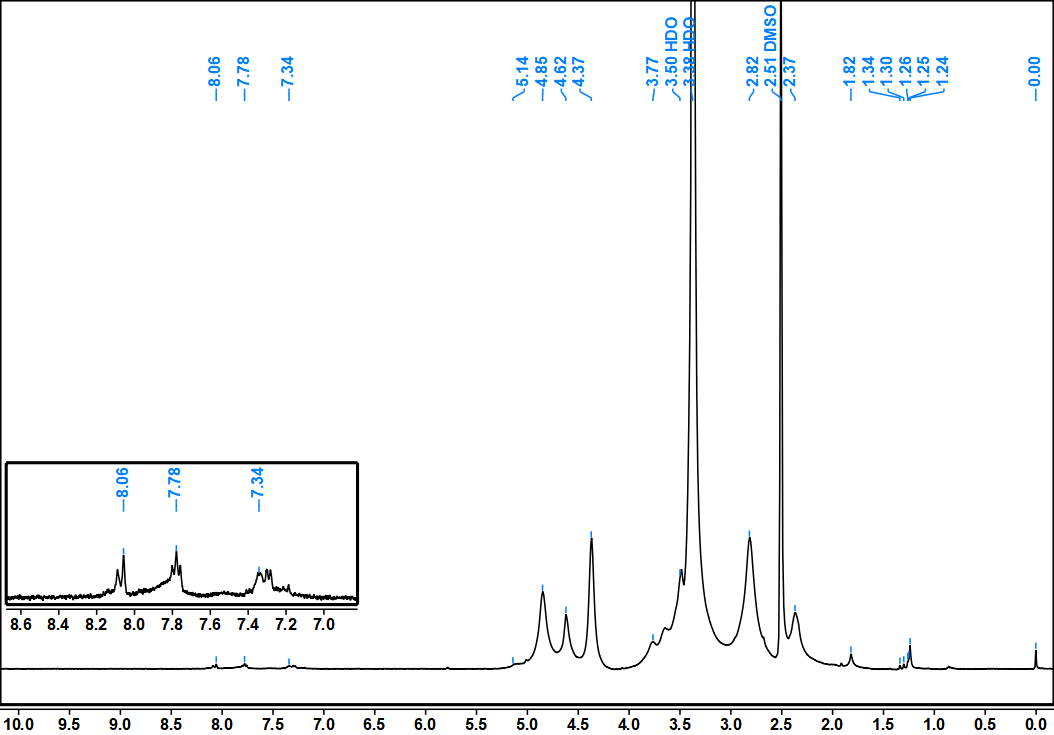


**Figure S3.** ^1^H NMR spectrum results of GcB.


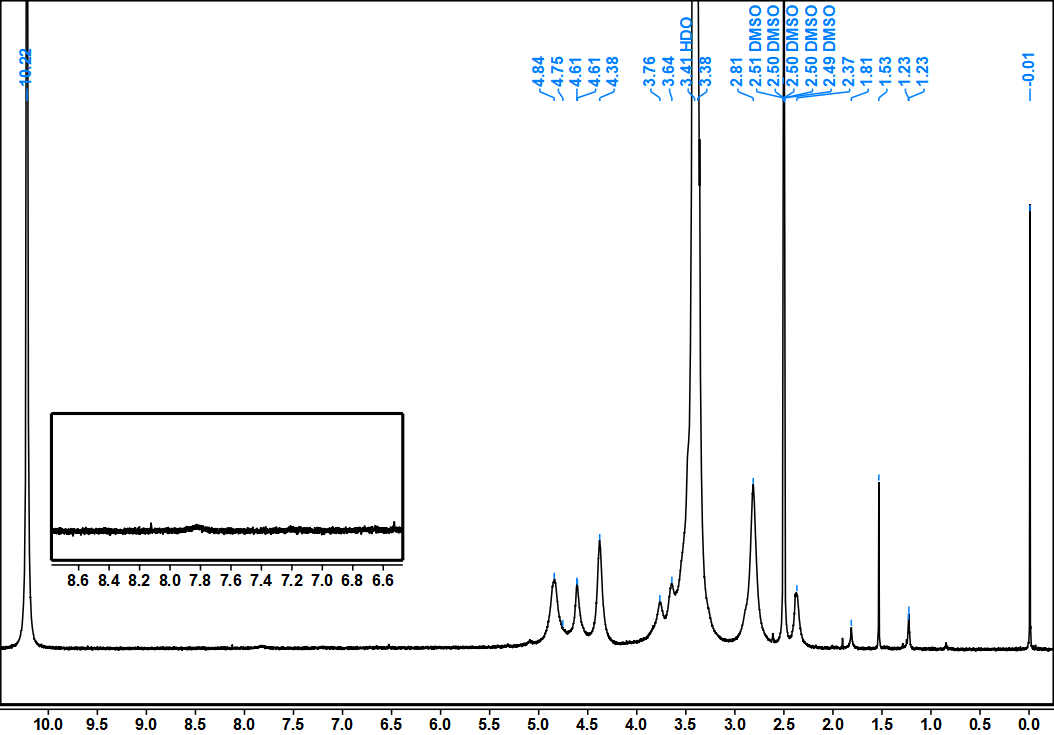


**Figure S4.** ^1^H NMR spectrum results for GcB treated with H_2_O_2_.


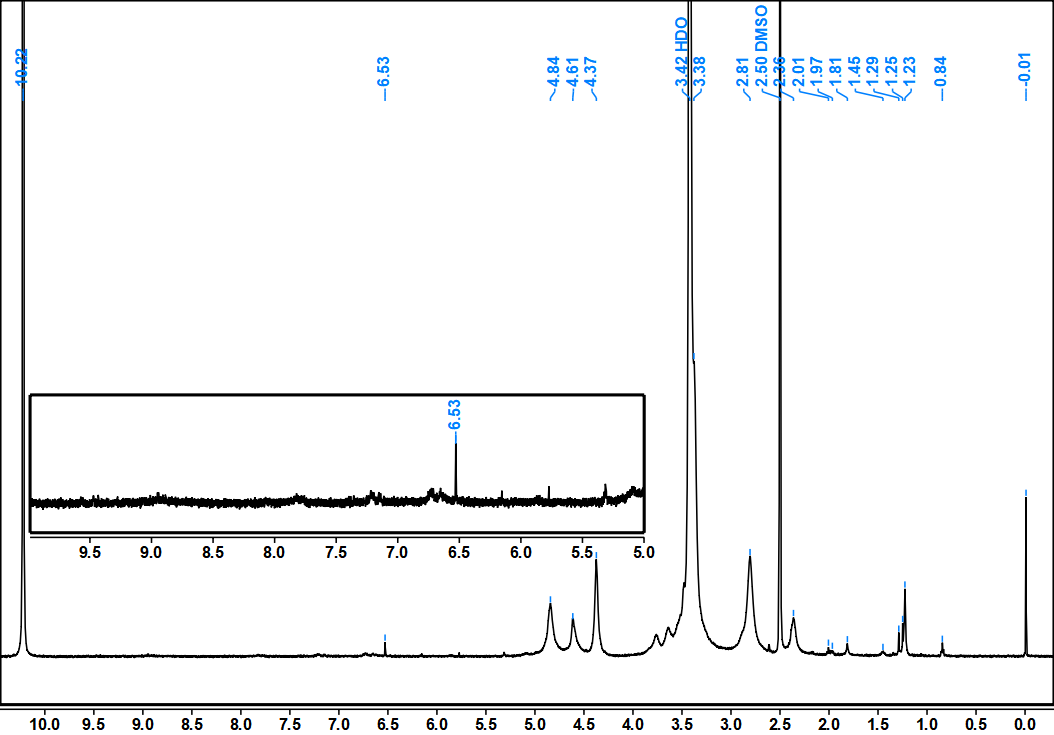


**Figure S5.** ^1^H NMR spectrum results of GcBC with H_2_O_2_ treated.


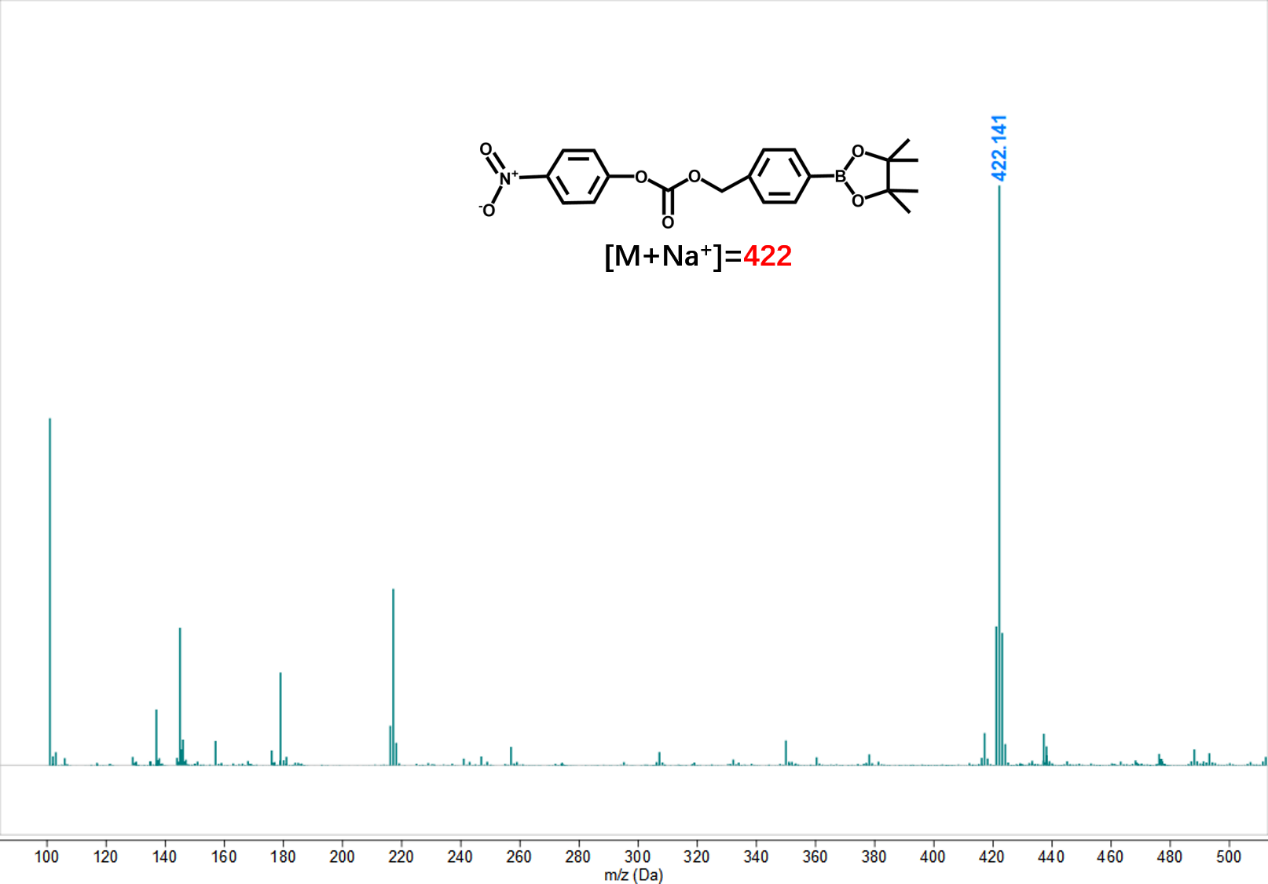


**Figure S6.** Mass spectrum results for phenylboronic acid ester (B).


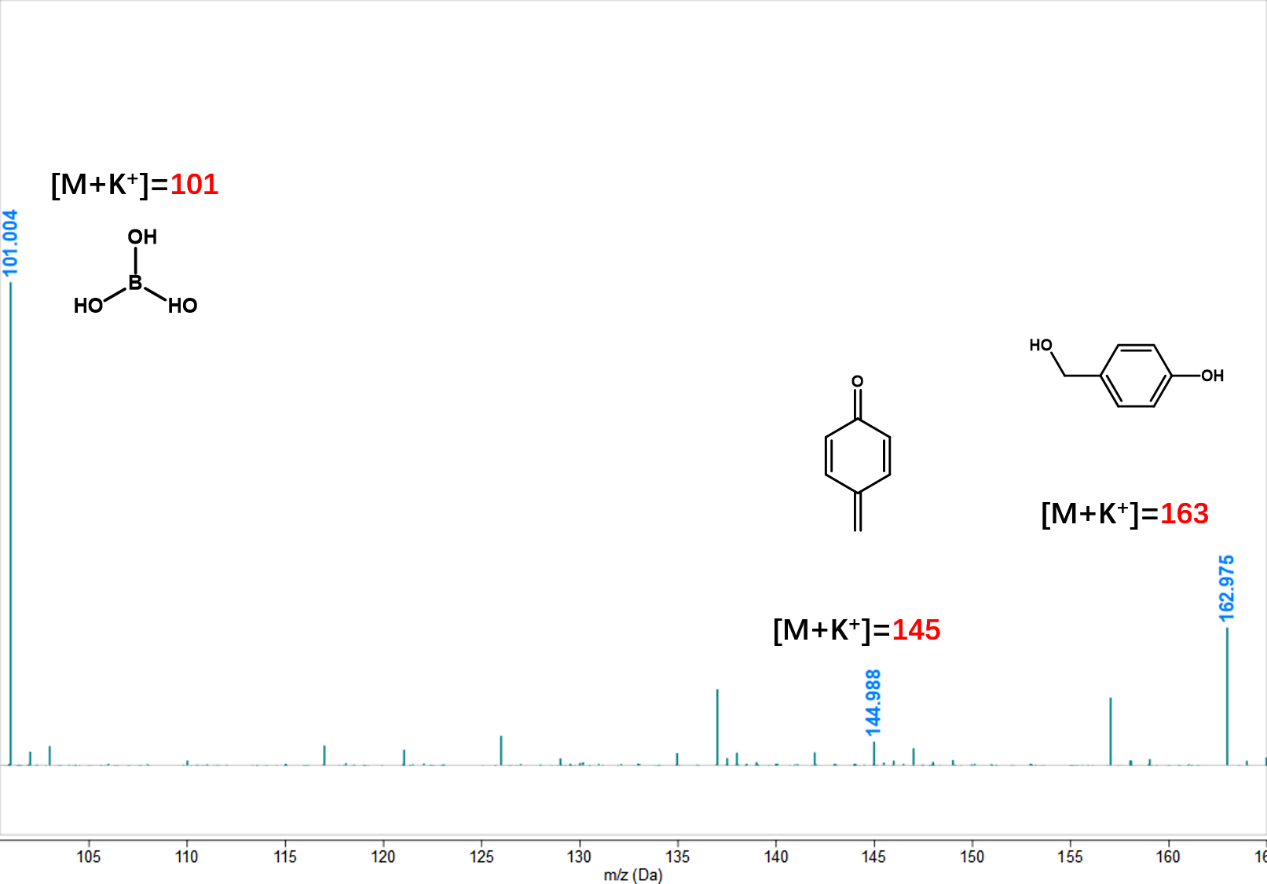


**Figure S7.** Mass spectrum results of GcB.

**
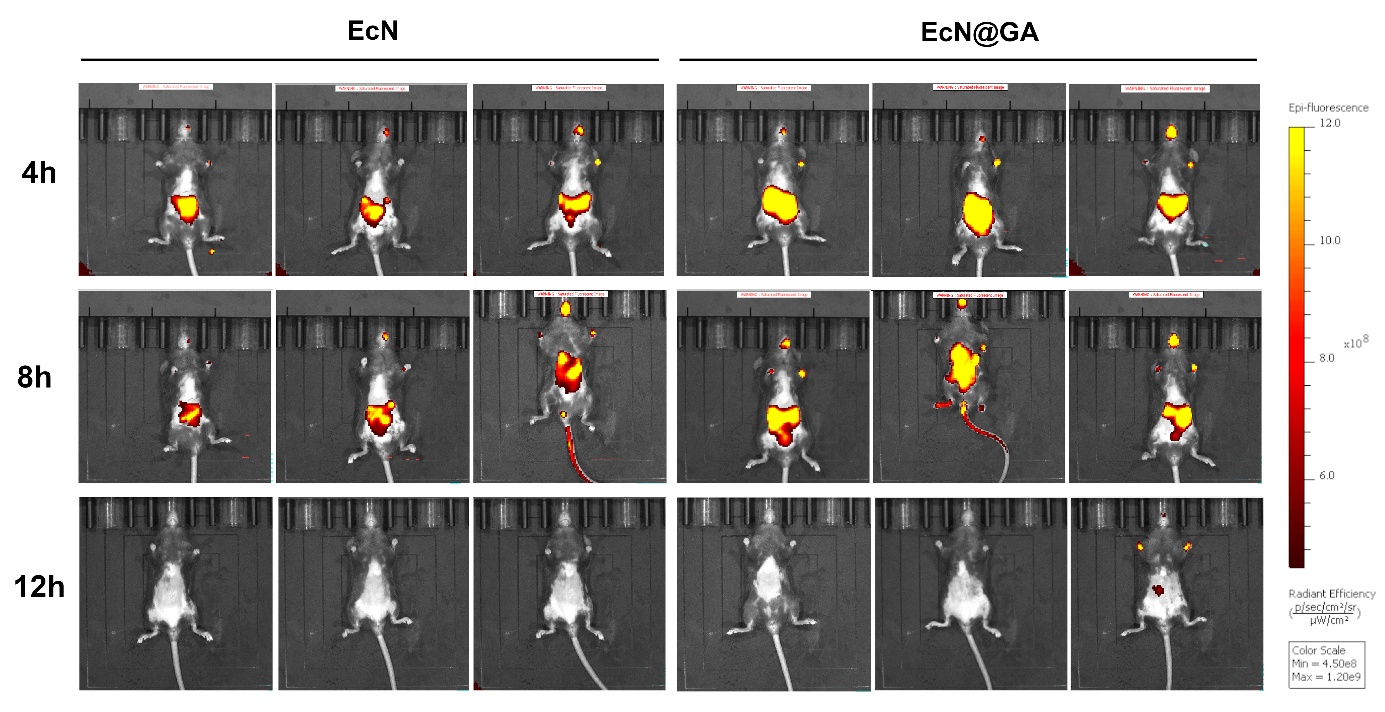
****Figure S8.** IVIS images of mice after oral administration of uncoated/coated EcN. Data are expressed as the mean ± standard error of the mean (SEM, n=3, biological replicates).


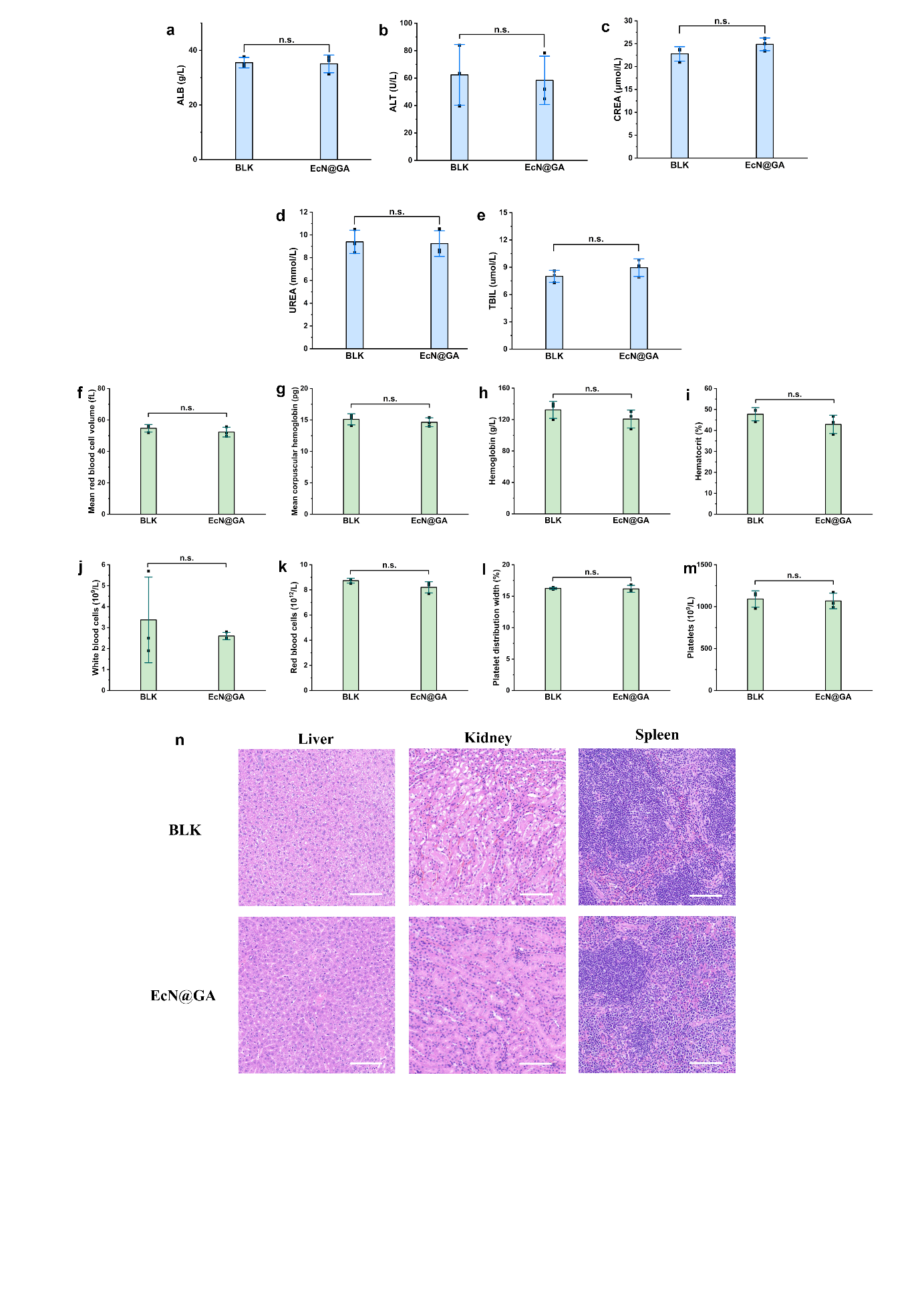


**Figure S9.** In vivo safety evaluation. **a**-**m**: Blood and serum biochemistry data for mice after the oral administration of PBS or EcN@GA for 7 days. ALB, albumin; ALT, alanine aminotransferase; CREA, creatinine; UREA, urea nitrogen; TBIL, total bilirubin. Data are expressed as the mean ± standard error of the mean (SEM, n=3, biological replicates), analyzed by one-way ANOVA, followed by Tukey’s test (n.s., not significant). **n**: representative H&E staining images of the liver, kidney, and spleen. scale bar: 100 μm
